# Analysis of the Glycosylation Profile of Disease-Associated Water-Soluble Prion Protein Using Lectins

**DOI:** 10.1155/2019/1053282

**Published:** 2019-02-11

**Authors:** Hanin Abdel-Haq

**Affiliations:** Department of Cell Biology and Neurosciences (Currently Department of Neurosciences), Istituto Superiore di Sanità, Viale Regina Elena, 00161 Rome, Italy

## Abstract

The disease-associated water-soluble form of hamster prion protein (ws-PrP^Sc^) has recently been found to be less stable than classical PrP^Sc^. Since the stability of PrP to degradation correlates with its glycosylation level, the aim of this study was to investigate whether there are differences between the glycosylation of ws-PrP^Sc^ and classical PrP^Sc^ of hamster which might account for the ws-PrP^Sc^ minor stability compared with that of the classical PrP^Sc^. Thus, ws-PrP and classical PrP were captured from noninfected or scrapie-infected hamster brain homogenate [high-speed supernatant (S^HS^) and high-speed pellet (P^HS^)] and blood plasma by anti-PrP antibodies (3F4 and 6H4) and subjected to screening for glycans by lectins under denaturing or nondenaturing procedures in a sandwich lectin-ELISA. Glycans have been found in minor quantities and differently exposed on ws-PrP^Sc^ from S^HS^ and plasma compared with classical PrP^Sc^ from P^HS^. These differences have been shown to be potentially responsible for the instability of ws-PrP^Sc^. Treatment of infected blood with GdnHCl significantly (P<0.01) increased the detection of ws-PrP^Sc^ in ELISA, reflecting an increase in its stability, and showed efficacy in removing high-abundance proteins in silver-stained gels. This increase in ws-PrP^Sc^ stability is due to an interaction of GdnHCl not only with high-abundance proteins but also with the ws-PrP^Sc^ glycosylation with particular regard to the mannose sugar. Analysis of lectins immunoreactivity toward total proteins from plasma collected before and at different time points after infection revealed that mannose might exert a stabilizing effect toward all of hamster blood glycoproteins, regardless of scrapie infection. Since low levels of ws-PrP^Sc^/soluble-infectivity have been estimated both in blood and brain of hamster, this glycosylation-related instability may have negatively influenced the propensity of ws-PrP^C^ to convert to ws-PrP^Sc^ both in blood and the brain. Therefore, PrP^C^ glycosylation characteristics may provide a tool for the determination risk of prion transmissibility.

## 1. Introduction

Transmissible spongiform encephalopathies (TSEs) or prion diseases are invariably fatal neurodegenerative diseases characterized by the conversion of the cellular prion protein (PrP^C^: classical PrP^C^) to the partially protease-resistant form (PrP^Sc^: classical PrP^Sc^, which is the hallmark of prion diseases) and its deposition in the central nervous system [1, 2].

A recent study revealed the existence of a water-soluble form of the prion protein (ws-PrP) in blood plasma and brain of Syrian hamster [3]. This PrP has biochemical-physical properties that are substantially different from those of the classical PrP. Particularly, a Western blot of normal ws-PrP (ws-PrP^C^) and disease-associated ws-PrP (ws-PrP^Sc^) [3] displayed a glycotyping that was different from that of the classical PrP^C^ and PrP^Sc^, showing a slightly faster migration mobility and a diglycoslated band with higher propensity to degradation by endogenous enzymes. This increased susceptibility to degradation of ws-PrP compared to the classical PrP may be due to an instability issue caused by glycosylation differences between the two proteins. Indeed, several sugars act as a stabilizing agent for proteins [4], and there is a correlation between glycosylation of proteins (in quantitative and qualitative terms) and their stability to enzymatic degradation. The oligosaccharide moiety is responsible for many glycoproteins' functions, such as synthesis, folding, trafficking, stability, recognition, and regulation of the proteins themselves and many of their diverse interactions [5, 6].

Therefore, glycosylation alteration is often accompanied by serious functional disorders such as prion diseases. In fact, glycosylation of prions appears to have considerable implications for the manifestations of disease [7]. Additionally, the location and composition of glycosylation contributed to the formation of various glycoforms of PrP^Sc^, giving rise to the different prion-strains and atypical glycoforms of PrP^Sc^ within one single prion strain [7]. Such glycoforms have been shown to contribute differentially to disease transmission, although the mechanism remains unclear.

Based on this relevant influence of the glycosylation on the formation of glycoforms of PrP with different properties, including the stability state, that are differentially associated with prion transmissibility, the aim of this study was to analyze the glycosylation profile of the water-soluble form of prion protein and classical PrP by using a panel of different lectins in ELISA, to investigate whether there are differences between the glycosylation of ws-PrP and classical PrP and whether such differences, if any, correlate with the ws-PrP minor stability in comparison to that of the classical PrP.

## 2. Materials and Methods

### 2.1. Preparation of the High-Speed Supernatant (S^HS^) Fraction

S^HS^ was prepared as described previously [8]. Briefly, brains from noninfected and terminally 263K-infected Syrian hamsters were homogenized, sonicated, and centrifuged at 825 x g for 15 min. Low-speed supernatant (S^LS^) was further ultracentrifuged at 220,000 x g for 30 min, yielding high-speed supernatant (S^HS^) and high-speed pellet (P^HS^). Protein content was determined by the bicinchoninic acid (BCA) protein assay (performed as the manufacture instructions, Sigma Chemical Co.). Hamsters used in this study were housed at the animal facility of the Istituto Superiore di Sanità under the supervision of the Service for Biotechnology and Animal Welfare of the institute, which adheres to national and international regulations and guidelines on animal welfare.

### 2.2. Preparation of Blood Plasma

Plasma was prepared as described previously [3, 9]. Blood was collected from individual Syrian hamsters (at time zero and at different time points after scrapie-infection) in tubes containing sodium citrate and separated into components by centrifugation at 2,000 x g for 15 min at 4°C. Collected plasma was stored at -80°C until analysis. Protein content was determined by the BCA protein assay.

### 2.3. Lectin-ELISA

96-well microtitre plates were loaded and incubated overnight at 4°C with equal amounts of total proteins from 263K-infected hamster plasma (collected at time zero and at different time points after infection) diluted in 50 *μ*l of carbonate buffer (pH 9.6) containing protease inhibitors cocktail (Complete, Roche Diagnostics, Mannheim, Germany). For sandwich lectin-ELISA, plates were coated overnight at 4°C with specific capture anti-PrP monoclonal antibodies 3F4 or 6H4 (Prionics) diluted in 50 *μ*l of carbonate buffer (pH 9.6) containing protease inhibitors. The coated wells were loaded with aliquots of sonicated 263K-infected S^HS^ (470 *μ*g total protein), P^HS^ (100 *μ*g total protein), or plasma (640 *μ*g total protein) and incubated overnight at 4°C. Wells were saturated in Tris-buffered saline containing 0.1% Tween-20 (TBS-T, pH 7.5) for 1 h at room temperature. Signals were developed by a sequential incubation with biotinylated lectins (10 *μ*g/mL of ConA, Concanavalin A; WGA, wheat germ agglutinin; RCA,* Ricinus communis* agglutinin; DBA,* Dolichos biflorus* agglutinin; PNA, peanut agglutinin; SBA, soybean agglutinin; and UEA-1,* Ulex europaeus* agglutinin I) (Vector Laboratories) for 2 h at room temperature, with a streptavidin horseradish peroxidase (HRP)-conjugated (2.5 *μ*g/mL) (Vector Laboratories) for 1 h at room temperature, and with an ABTS Substrate (Vector Laboratories) for 20 min in the dark at room temperature. Then, optical density of the developed color was measured at 405 nm.

**Figure 1:**
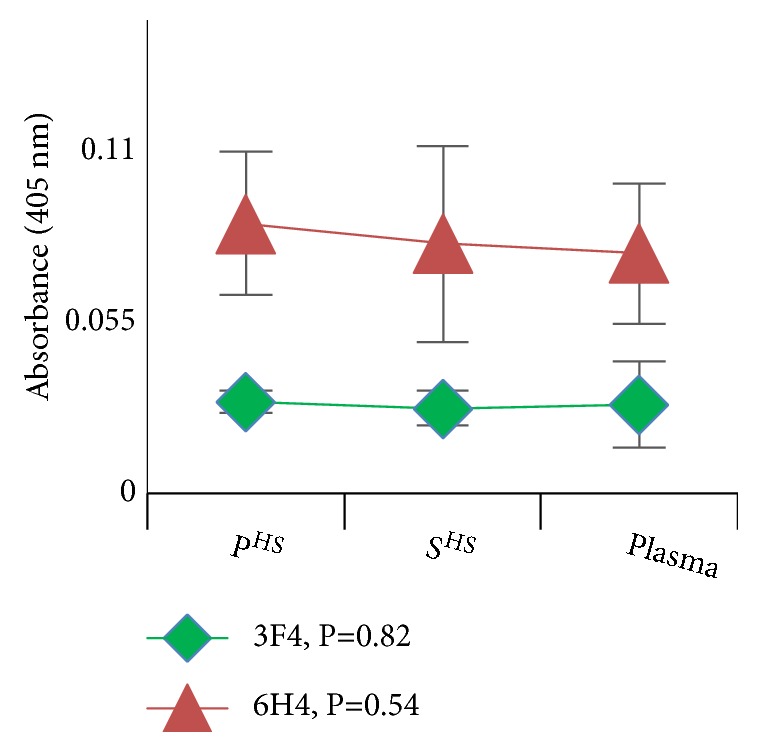
*The binding of the PrP to the capturing antibodies was saturated*. After sonication, an excess of total proteins from P^HS^, S^HS^, and plasma, calculated in order to achieve a saturated binding of PrP to the coated antibodies, was loaded into 96-well ELISA plates that were precoated with the anti-PrP capturing antibody. The figure shows similar absorbance values for PrP from P^HS^, S^HS^, and plasma indicating that equivalent amounts of PrP were captured and then identified by the antibodies. Since similar amounts of PrP were captured by 3F4 and 6H4 antibodies in the sandwich lectin-ELISA, this figure serves to support that the minor quantity of sugars detected on ws-PrP with respect to classical PrP is not due to the capturing of a minor amount of ws-PrP, but it rather reflects a difference in lectin binding. Data are means ± SD and are representative of at least three independent assays and three different preparations, performed in duplicate.

The amount of total proteins from plasma, S^HS^, and P^HS^ loaded into ELISA plate is based on calculations made in a previous study [3], in which PrP^Sc^ from plasma, S^HS^, and P^HS^ had been quantitatively and qualitatively characterized. These amounts of total proteins (which contain an excess of PrP^Sc^ that guarantee a saturated binding to the antibodies) have been calculated in order to allow for the binding of equivalent amounts of PrP to the capturing antibodies, as shown in Figure 1. Thus, the differences in ELISA readings would not be due to quantitative difference in the amounts of captured PrP but would rather reflect the difference in lectin binding.

### 2.4. Lectin Blotting

Total proteins from uninfected and 263K-infected plasma (terminal stage) were subjected to electrophoreses on 10% polyacrylamide gel and blotting onto nitrocellulose membrane as described previously [10]. The membranes were blocked in TBS-T for 1 h at room temperature and then incubated with biotinylated-lectins (ConA, WGA, RCA, DBA, PNA, SBA, UEA-1) diluted in TBS-T at a final concentration of 1 *μ*g/mL for 2 h at room temperature. After washing six times with TBS-T, membranes were incubated with a streptavidin HRP-conjugated diluted in TBS-T for 1 h at room temperature. Signals were visualized on X-ray film (Amersham) by using the Enhanced Chemiluminescence (ECL) detection system (SantaCruz).

### 2.5. ELISA

Precoated wells with a specific capture anti-PrP antibody were loaded with the plasma and IgG samples treated or not treated with GdnHCl (at ratios of 1:1 or 1:2 as stabilizing agent), as below described, and incubated overnight at 4°C. Wells were rinsed quickly with Tris-buffered saline containing 0.015% Tween 20 (TBS-T) and saturated with TBS-T containing 3.3% nonfat dry milk for 30 min at room temperature. Then, they were incubated with the anti-PrP monoclonal antibody 6H4 for 1 h at 37°C with mild shaking. Signals were measured after a sequential incubation with an HRP-conjugated secondary antibody for 1 h at 37°C with mild shaking, and with chemiluminescent HRP substrate (Roche) for 10 min at room temperature with shaking.

### 2.6. Silver Staining

12% SDS-PAGE gels loaded with plasma total proteins treated or not treated with GdnHCl (at a ratio of 1:2 as stabilizing agent), as below described, were fixed in 50% methanol/12% acetic acid/0.0185% formaldehyde overnight or for at least 1 h at room temperature with shaking. Gels were washed in 50% (2×20 min) and 30% ethanol (1×20 min), respectively. Then, gels were reduced in 0.02% sodium thiosulfate solution (1 min), washed with bidistilled water (3×20 sec), and exposed to 0.2% silver nitrate/0.0185% formaldehyde (30 min). After a brief wash with bidistilled water (2×20 sec), gels were incubated in developing solution (3% sodium carbonate/0.028% formaldehyde/0.0004% sodium thiosulfate) until bands appearance and stopped by washing with bidistilled water (2×1 min). Stained gels were then incubated with 50% methanol/12% acetic acid (10 min) and washed with 50% methanol (20 min) before being scanned to acquire the image.

### 2.7. Treatment with Guanidine Hydrochloride (GdnHCl)

#### 2.7.1. GdnHCl as Denaturing Agent

Treatment with GdnHCl was performed as described previously [9]. 4 M GdnHCl (Sigma) was added to the samples before loading (ratio of 1:1 (v/v)). Treated samples were heated for 10 min at 100°C with shaking. Then, they were brought with phosphate-buffered saline (PBS) to the requested dilution.

#### 2.7.2. GdnHCl as Stabilizing Agent

Fixed volumes of hamster plasma were incubated with different volumes of 4 M GdnHCl for 20 min at 65°C with shaking. Samples were centrifuged at 0.5 x g for 1 min at room temperature and supernatants were treated with ice cold methanol at a ratio of 1:2 (v supernatant: v methanol) to induce the precipitation of GdnHCl residual from the samples and then centrifuged again at 0.5 x g for 30 seconds at room temperature. Supernatants were concentrated by centrifugal evaporation (SpeedVac, Savant) and pellets were resuspended in PBS (pH 7.4) for ELISA or in sample buffer for SDS-PAGE. Different ratios between plasma and GdnHCl (v GdnHCl: v plasma = 1:10, 1:5, 1:3, 1:2 or 1:1) were tested and the maximum effect was achieved with the ratio of 1:2.

### 2.8. Preparation of IgGs

Hamster plasma pretreated with 1 M Tris HCl (pH 8) (1 part of Tris and 10 parts of plasma) was depleted from IgGs by saturation with saturated ammonium sulfate solution at a ratio of 1:1 (v:v) for at least 1h at 4°C with slight mixing. The IgGs were precipitated by centrifugation at 10.000 x g for 20 min at 4°C and the obtained pellet was resuspended with a volume of water equal to the plasma initial volume.

### 2.9. Statistical Analysis

Percentages were calculated from the ratio between the measured value and the reference value, multiplied per 100. Statistical differences between groups were evaluated by student's t-test and P < 0.05 was considered statistically significant. The arbitrary units were calculated by normalization of the measured values against the reference values. Data represent mean ± SD.

## 3. Results and Discussion

Classical PrP^C^ and PrP^Sc^ have a similar glycosylation profile, although some glycosylation differences between them have been detected [9, 11], and ws-PrP^C^ and ws-PrP^Sc^ have identical biochemical-physical properties [3]. Taking into account these two characteristic aspects of the classical PrP and ws-PrP, the comparisons between the two proteins were performed using infected material and only when necessary also noninfected material. The use of infected material may allow for achieving new insights on the pathological mechanism while providing information about the glycans content and exposure on classical and water-soluble PrP.

### 3.1. Glycosylation Profile of ws-PrP^Sc^ and Effect of GdnHCl on Its Stability

To investigate the glycosylation profile of ws-PrP^Sc^, brain-derived and plasma-derived ws-PrP^Sc^ was captured from terminally scrapie-infected hamster high-speed supernatant (S^HS^) and plasma, respectively, by 3F4 or 6H4 anti-PrP monoclonal antibodies and then subjected to screening for glycans by a panel of seven biotinylated lectins in a sandwich lectin-ELISA.

**Figure 2:**
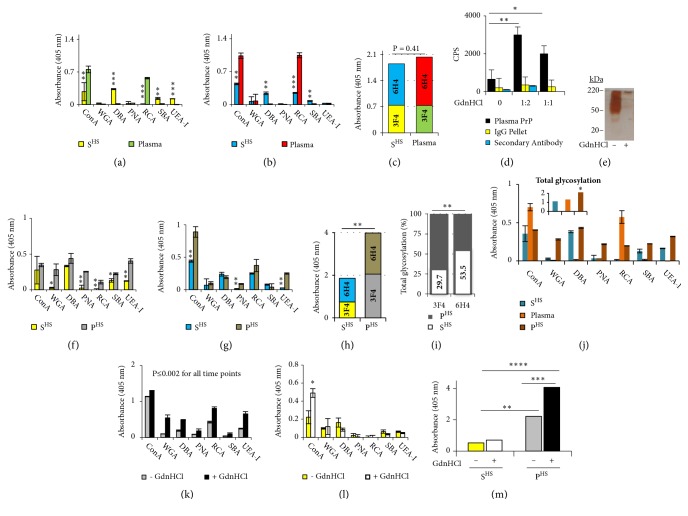
*Analysis of the glycosylation profile of ws-PrP and classical PrP and effect of treatment with GdnHCl*. ((a)-(c)) Glycosylation profile of PrP^Sc^ from high-speed supernatant (S^HS^) and plasma is similar: ((a) and (b)) lectin-binding profile to single glycans in PrP^Sc^ captured by 3F4 and 6H4, respectively, from S^HS^ and plasma; (c) glycans total content in PrP^Sc^ captured by 3F4 and 6H4 from S^HS^ and plasma. (d) Comparison between signals of PrP^Sc^ captured by specific anti-PrP polyclonal antibody from 263K-infected hamster plasma and signals of immunoglobulin (IgG) prepared from 263K-infected hamster plasma, before and after treatment with guanidine hydrochloride (GdnHCl) in ELISA. Signals were detected by 6H4 antibody/secondary antibody or only with secondary antibody. (e) Representative silver-stained 12% SDS-PAGE gel of hamster plasma total proteins showing effect of GdnHCl on high-abundance proteins removal from blood. ((f)-(m)) Glycosylation profile of ws-PrP differs from that of classical PrP: ((f) and (g)) lectins binding profile to single glycans in PrP^Sc^ captured by 3F4 and 6H4, respectively, from S^HS^ and high-speed pellet (P^HS^); (h) glycans total content in PrP^Sc^ captured by 3F4 and 6H4 from S^HS^ and P^HS^; (i) percentage of glycans total content in S^HS^  PrP^Sc^ with respect to that in P^HS^PrP^Sc^; (j) lectins binding profile to glycans in PrP^Sc^ captured by 3F4 from noninfected S^HS^, plasma and P^HS^; ((k) and (l)) lectins binding profile to single glycans in PrP^Sc^ captured by 3F4 from P^HS^ (k) and S^HS^ (l) before and after treatment with GdnHCl; (m) glycans total content in PrP^Sc^ captured by 3F4 from S^HS^ and P^HS^ before and after treatment with GdnHCl. Total proteins from plasma, S^HS^ and P^HS^ were loaded into 96-well ELISA plates that were precoated with the 3F4 or 6H4 antibodies, and the lectin-binding profile was determined by staining with ConA, Concanavalin A; WGA, wheatgerm agglutinin; RCA,* Ricinus communis* agglutinin; DBA,* Dolichos biflorus* agglutinin; PNA, peanut agglutinin; SBA, soybean agglutinin; and UEA-1,* Ulex europaeus* agglutinin I. The sizes of molecular mass markers in kilodaltons are indicated on the left. Data are means ± SD and are representative of at least three independent assays and three different preparations, performed in duplicate. *∗* P<0.05, *∗∗* P<0.01, *∗∗∗* P<0.001, *∗∗∗∗* P<0.0001.

Plasma and S^HS^  PrP^Sc^ were recognized by all of the lectins, showing a similar glycosylation pattern, but different proportions of the individual sugars, particularly those recognized by Concanavalin A (ConA) and* Ricinus communis agglutinin* (RCA) (Figures 2(a) and 2(b)). The much higher reactivity of these two lectins could be due to a major abundance or exposure of the specific sugars due to a functional importance, but not to the PrP^Sc^ amount since PrP^Sc^ was captured by an equal quantity of antibodies from plasma and S^HS^. However, the overall content in sugars of S^HS^  PrP^Sc^ did not differ from that of plasma PrP^Sc^ (P = 0.41) and was shown to be significantly (P<0.05) increased in both S^HS^ and plasma PrP^Sc^ when PrP was captured by 6H4 in comparison to when it was captured by 3F4 (Figure 2(c)).

Because guanidine hydrochloride (GdnHCl) can stabilize proteins [12], to evaluate its effect on ws-PrP^Sc^ stability and to explore the possible relationship between glycosylation profile of ws-PrP^Sc^ and its stability to enzymatic degradation, 263K-infected hamster plasma was treated with 4 M GdnHCl at different ratios as described in materials and methods. Treatment of plasma samples with GdnHCl not only increased the stability of ws-PrP^Sc^, improving its detection, but it also inhibited the interference exerted by the IgGs toward low-abundance proteins, as is clearly shown in Figure 2(d). Figure 2(d) shows PrP^Sc^ signals from plasma and signals from IgG preparation detected in ELISA before and after treatment with GdnHCl at ratios 1:1 and 1:2. In blood, IgGs and high-abundance proteins in general have shown to compete with low-abundance proteins, including PrP, reducing the sensitivity and specificity of detection assays either by masking the presence of PrP^Sc^ or by interacting directly with the detection antibodies [13].

At both the treatment ratios 1:1 and 1:2, the signal of PrP^Sc^ significantly (P≤0.05 and P<0.01 at 1:1 and 1:2, respectively) increased compared to before treatment and also compared with the signal of the IgG before and after treatment with GdnHCl (P≤0.01 and P<0.001 at 1:1 and 1:2, respectively). The signal of PrP^Sc^ at a ratio of 1:2 was considerably higher than at the 1:1 ratio (P = 0.07, 33.3%), while the signal of the IgG at a ratio of 1:2 did not significantly differ from the signal at a ratio of 1:1 and before treatment (Figure 2(d)). Signals obtained by detection with the secondary antibody omitting the primary were comparable to those of the IgG signals (P = 0.476) and are shown in Figure 2(d). These results suggest that GdnHCl is able to increase the stability of the ws-PrP present in blood, and it may do this besides by an interaction with the interfering IgGs also by an interaction with the glycosylation profile of ws-PrP.

Treatment with GdnHCl also showed efficacy in removing high-abundance proteins, as shown in silver-stained gels.  Figure 2(e) shows a consistent reduction in the extent and intensity of the signal (silver stain) of high-abundance proteins after treatment with GdnHCl as compared with the signal before treatment.

This removal capacity of GdnHCl may be due to the fact that the heating of the sample induces the coagulation of some plasma proteins including IgG and albumin and releases low-abundance proteins in the aqueous fraction. Otherwise, denaturing with GdnHCl may break the bond between low- and high-abundance proteins, causing the release of low-abundance proteins in the aqueous fraction. In agreement with these explanations in presence of GdnHCl, the yield of low-abundance proteins that are enriched in the aqueous fraction and can be separated by centrifugation was significantly greater than without treatment. This finding is an important added value for the field of the biomarker discovery using blood: by providing an enriched source of low-abundance proteins including PrP, which are usually more involved in pathological processes than high-abundance proteins, and by reducing or even eliminating the interfering action of high-abundance proteins, avoiding thus confused and artifact results.

One possible mechanism for the stabilizing action of GdnHCl to the benefit of low-abundance-proteins detection in blood is that GdnHCl has the capacity to denature proteins including albumin and IgG by changing their conformation and consequently altering their normal function, such as their ability of binding to some ligands [14]. Such conformational alteration of proteins' binding sites exerted by GdnHCl would overwhelm the interfering IgGs and other high-abundance proteins, reducing the probability that they will bind to the detector or capture antibodies and increasing instead the probability of detecting low-abundance proteins in blood. Similarly, a conformational alteration of binding sites of protein-degrading enzymes would preserve several proteins including PrP^Sc^ from degradation increasing thus their stability and detection in blood. Also, as consequence of conformational alteration of proteins' binding sites, the exposure of some glycans (one or more) on some glycoproteins may change in a manner that they become more exposed than others. This increased exposure of some glycans would in turn increase the masking of the vulnerable cleavage sites increasing thus the stability of proteins to enzymatic degradation.

Thus, the increased detection of ws-PrP in blood after treatment with GdnHCl (increased stability) may be also attributed to alteration in the exposure of distinct glycans on ws-PrP, suggesting that GdnHCl may stabilize proteins through an interaction with their glycosylation. In light of this latter suggestion about the mechanism of proteins stabilization by GdnHCl, the *α*–linked mannose and the N-acetylgalactosamine glycans recognized by ConA and RCA, respectively, which have been distinguished with respect to the other tested glycans as shown in Figures 2(a) and 2(b), could be potential candidates glycans to be involved in the ws-PrP stabilization process.

These results collectively suggest that GdnHCl at the same applied concentration explicates a stabilizing effect on some blood proteins including PrP and causes destabilization of other proteins such as enzymes, IgGs, and albumin.

### 3.2. Comparison between the Glycosylation Profile of ws-PrP and Classical PrP and Effect of Treatment with GdnHCl on the Glycans Exposure

To investigate whether there are differences between the glycosylation profile of ws-PrP^Sc^ and classical PrP^Sc^ that may be responsible for the minor stability of ws-PrP^Sc^, S^HS^ (source of ws-PrP^Sc^) and high-speed pellet (P^HS^) (source of classical PrP^Sc^) from a scrapie-infected hamster brain homogenate were subjected to screening for PrP^Sc^ glycans in a sandwich lectin-ELISA, as above described. When PrP^Sc^ was captured by the 3F4 antibody, S^HS^  PrP^Sc^ showed a glycosylation pattern similar to that of P^HS^  PrP^Sc^ but displayed minor proportions of sugars (Figure 2(f)). When PrP^Sc^ was captured by the 6H4 antibody, except for an increase in the immunoreactivity of RCA and ConA, the immunoreactivity of all of the lectins was reduced toward both S^HS^ and classical PrP^Sc^ (Figure 2(g)). This reduction was consistent for the classical PrP^Sc^ but not for the ws-PrP^Sc^ (Figure 2(g)). The reason of this apparent discrepancy between the two antibodies is not clear. However, based on a similar result from an earlier study, in which antibodies partially blocked lectin binding to the PrP glycans due to the close location of the antibodies epitopes to the glycosylation sites [15], it may be due to the fact that the glycan attachment sites are located in the C-terminal region of the PrP at amino acids 181 and 197 (in the hamster sequence), which are in close proximity to the 6H4 epitope (C-terminal antibody, aa 144-152) and distant from the 3F4 epitope (N-terminal antibody, aa 109-112). It has been reported that the flexibility of the PrP in the C-terminal region is lower than in the N-terminal region and that this effect is induced by the N-linked glycans themselves [5]. Therefore, this different immunoreactivity of the lectins in presence of the C-terminal and N-terminal capturing antibodies could be correlated with the flexibility state of the PrP in the respective regions. This negative relationship between 6H4 C-terminal antibody and immunoreactivity of lectins is not totally unexpected since besides the aforementioned example [15] others are reported in the literature [16]. This different influence of the capture antibody on PrP glycans exposure was less observed between S^HS^ and plasma PrP^Sc^, indicating a structural similarity between them and consequently a similar glycosylation profile (Figures 2(a), 2(b), and 2(c)).

Overall, results indicate that ws-PrP^Sc^ has a glycosylation profile that is substantially different from that of the classical PrP^Sc^. This difference could be responsible for ws-PrP^Sc^ minor stability.

ws-PrP^Sc^ content in glycans is also consistently lower than the content of classical PrP^Sc^ for several reasons: (1) the overall content of glycans in S^HS^  PrP^Sc^ is significantly (P<0.01) lower than in P^HS^  PrP^Sc^ (Figures 2(h) and 2(i)); (2) using 2 different specific monoclonal anti-PrP antibodies recognizing N-terminal epitopes (3F4) and C-terminal epitopes (6H4) allowed the capturing of both full length and fragments of PrP, assuring that all the PrP molecules present in the sample are captured and detected by the lectins; (3) a standard quantity of antibody was loaded to capture an identical quantity of ws-PrP^Sc^ and classical PrP^Sc^ from an excess amount of total proteins (saturated binding, see Materials and Methods); (4) a similar difference in the glycans content between the classical and water soluble glycoforms of PrP from noninfected P^HS^, S^HS^, and plasma was observed (Figure 2(j)), confirming the existence of such a difference also at the level of the cellular PrP (before its conversion to PrP^Sc^), which clearly indicates that it has a physiological origin; (5) to avoid the capturing of large aggregates of PrP, S^HS^, P^HS^, and plasma were subjected to sonication before loading into ELISA plate.

To both rule out that this minor content of sugars in ws-PrP^Sc^ may be merely due to exposure or conformational alterations and to assess the effect of GdnHCl on glycans exposure on both ws-PrP^Sc^ and classical PrP^Sc^, S^HS^  PrP^Sc^, and P^HS^  PrP^Sc^ were denatured with 4 M GdnHCl. Denaturation with GdnHCl significantly increased the immunoreactivity of all of the lectins toward the classical PrP^Sc^ (P≤0.002, Figure 2(k)) but not toward S^HS^  PrP^Sc^, where only immunoreactivity of ConA increased significantly (P<0.05, Figure 2(l)). This result suggests that after treatment with GdnHCl (compared to before treatment), the exposure of glycans present on wsPrP^Sc^ remained almost unchanged, while the exposure of glycans present on classical PrP^Sc^ was subjected to substantial improvement. This different behavior between the glycosylation of S^HS^ and classical PrP^Sc^ to the treatment with GdnHCl may be due to the fact that the 3F4 epitope is less buried in ws-PrP^Sc^ than in the classical PrP^Sc^ [3]. This also implies that the glycosylation on ws-PrP^Sc^ is less masked. Consequently, the overall content in sugars of S^HS^PrP^Sc^ did not significantly change after treatment with GdnHCl (P = 0.289), while the overall content in sugars of classical PrP^Sc^ significantly increased both compared to before denaturation (P≤0.001, 83%) and compared with S^HS^  PrP^Sc^ (P≤0.0001, 82.76%) (Figure 2(m)). These results unequivocally demonstrate and further support that the ws-PrP^Sc^ content in glycans is lower and more exposed than that of the classical PrP^Sc^. The S^HS^  PrP^Sc^ total content in glycans is 4.22-fold (P≤0.01) and 3.37-fold (P<0.00005) lower than that of P^HS^  PrP^Sc^ before treatment and after treatment with GdnHCl, respectively. It is worth noting that even before denaturation and subsequent uncovering of 3F4 epitope, the classical PrP^Sc^ content in glycans was significantly (P<0.01) higher than that of ws-PrP^Sc^ (76.3%* vs *23.7%) (Figure 2(m)). It is known that 3F4 epitope is always exposed in native classical PrP^C^ and becomes buried after conversion to the native classical PrP^Sc^ [17]. Considering that under native conditions (before treatment with GdnHCl), the immunoreactivity of PrP^Sc^ is similar to that of PrP^C^ but under denaturing conditions (after treatment with GdnHCl), the immunoreactivity of PrP^Sc^ markedly increases with respect to that of PrP^C^, which does not significantly change [17]; this characteristic detail of PrP^Sc^ has often been exploited by researchers to distinguish between PrP^C^ and PrP^Sc^ at the native state in conformational assays using GdnHCl.

Such a glycosylation profile confers instability to ws-PrP^Sc^ and makes it more vulnerable to different kinds of insults than classical PrP^Sc^. Indeed, a protective role of the glycosylation against proteolytic agents by rendering vulnerable cleavage sites of PrP (and other proteins) inaccessible to their action has been known [5, 18]. This glycosylation profile of ws-PrP^Sc^ in comparison to that of the classical PrP^Sc^ may be responsible for its increased sensitivity to digestion by proteinase K and its smaller molecular mass, which both have previously been revealed as characteristics for ws-PrP^Sc^ [3]. Indeed, a reduced content of glycans would entail a reduction both in the molecular weight of ws-PrP^Sc^ and in the protection for its cleavage sites.

On the basis of these logical and sequential connections, the glycosylation profile of ws-PrP^Sc^ could be directly correlated with its instability state. Furthermore, results support the earlier supposition about a potential interaction of GdnHCl with the *α*–linked mannose glycan, recognized by ConA, in order to stabilize ws-PrP, confirming thus that this glycan could be indeed relevant for the stability of ws-PrP. Additionally, considering that the classical PrP^Sc^ is much more stable than the ws-PrP^Sc^, it seems that the number of glycans whose exposure has been altered on the ws-PrP^Sc^ and classical PrP^Sc^ by GdnHCl is positively correlated with the final stability state of the single proteins.

### 3.3. Role of *α*–Linked Mannose Glycan in the Stability of Plasma Total Proteins

**Figure 3:**
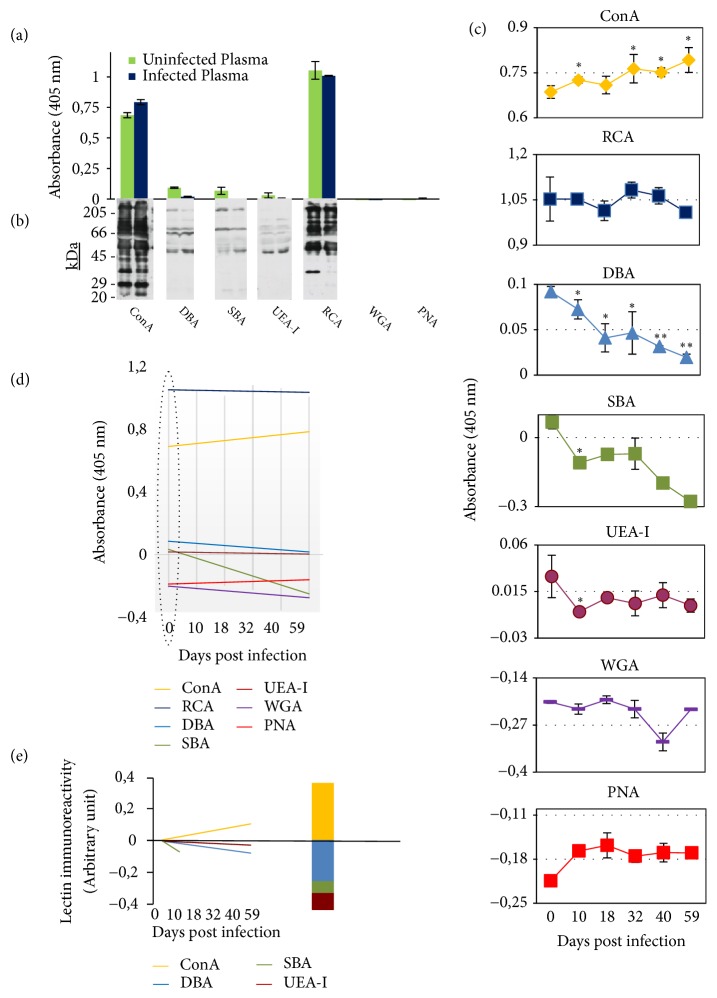
*Glycosylation profile of hamster plasma total proteins is essential for their stability in blood*. ((a)-(c)) Binding profile of ConA, Concanavalin A; WGA, wheatgerm agglutinin; RCA,* Ricinus communis* agglutinin; DBA,* Dolichos biflorus* agglutinin; PNA, peanut agglutinin; SBA, soybean agglutinin; and UEA-1,* Ulex europaeus* agglutinin I to equal amounts of total proteins from noninfected and terminally scrapie-infected hamster plasma ((a) and (b)) and from scrapie-infected hamster plasma collected at different time points after infection (c). ((a) and (c)) Lectin-ELISA analysis, (b) lectin-blot analysis (10% SDS-PAGE gel). The figures show that lectins binding profile to plasma total proteins is not due to the infection or disease duration but rather to potential relevance of the glycans recognized by ConA and RCA for maintaining the stability of plasma total proteins including PrP against certain insults. (d) Comparison between the regression lines of the binding profile of ConA, WGA, RCA, DBA, PNA, SBA, and UEA-1 toward plasma total proteins throughout disease period. The basal immunoreactivity of ConA and RCA is the highest and this trend is maintained also throughout disease period; only immunoreactivity of ConA (increase) and DBA (decrease) correlates with disease progression (r = 0.90); lectins basal immunoreactivity values are potential predictors of the expected effect by the relative sugars on proteins stability state. (e) The increase in the immunoreactivity of ConA is proportional to the sum of reduction in the immunoreactivity of DBA, SBA, and UEA-I. The left side of the figure shows that the two effects (decrease and increase of immunoreactivity) are inversely correlated between each other during disease progression, while the right side of the figure shows that the two effects (decrease and increase of immunoreactivity) are mathematically very close but opposite. The increase and decrease in the lectins reactivity were calculated by normalization of the absorbance values against the basal reactivity values for each lectin. Lectins basal reactivity is indicated by a circle shown on Figure 3(d). The sizes of molecular mass markers in kilodaltons are indicated on the left. Data are means ± SD and are representative of at least three independent assays and three different preparations, performed in duplicate. *∗* P<0.05, *∗∗* P<0.01.

Because *α*–linked mannose glycan in comparison to other tested glycans appears potentially involved in the stabilization process of the ws-PrP^Sc^, it has been attempted to investigate this issue and to assess whether this glycan contributes to the stability of the ws-PrP in blood in a selective and specific manner. Thus, total proteins from terminally infected plasma were analyzed by lectin-ELISA. Analysis revealed a lectin binding profile toward the glycosylation of plasma total proteins very similar to that previously observed toward the glycosylation of the PrP (Figure 3(a)). This result suggests that this profile, characterized by highest reactivity of the sugars recognized by ConA and RCA, is not specific for the PrP but is distinct for all the plasma glycoproteins and that such a profile seems to be essential. Additionally, this particular lectin binding profile revealed not to be induced by the scrapie infection since it was observed also for the glycosylation of total proteins from noninfected plasma (Figure 3(a)), regardless of the glycosylation alterations between infected and noninfected plasma total proteins [19]. Such lectin binding profile toward the glycosylation of total proteins from noninfected and terminally infected plasma was also observed on lectin-blot (Figure 3(b)). These results support, once more again, the idea about the existence of a functional relevance for the *α*–linked mannose and the N-acetylgalactosamine glycans recognized by ConA and RCA, respectively, and suggest that they might be involved in maintaining the stability of glycoproteins including PrP, present in the blood plasma.

To explore the possible mechanism by which these two sugars act to maintain stability of plasma proteins* in vivo*, equal amounts of total proteins from scrapie-infected hamster plasma, collected at time zero and at different time points after infection, were subjected to lectin-ELISA. Analysis of lectins reactivity in first place confirmed the typical lectin binding profile toward the plasma glycoproteins glycosylation and secondly revealed that the *α*–linked mannose glycan recognized by ConA appears to be the only glycan, within the tested glycans, that might be involved in the stabilization process of glycoproteins in plasma after infection. In fact, ConA immunoreactivity was increased soon after infection and maintained this trend throughout the course of the disease in a linear relationship (P<0.05, r = 0.84) (Figure 3(c)), while the immunoreactivity of RCA did not change significantly (Figure 3(c)). This increase of the *α*–linked mannose glycan, which is correlated with disease progression* in vivo*, might have been required by the homeostasis as a tentative to counterbalance the drastic and marked decrease in the amount or exposure of the glycans recognized by SBA, UEA-I, and DBA (~45%, 40% and 21.2 %, respectively) at 10 days after infection (Figure 3(c)). Particularly, in Figure 3(c) it is clearly shown how the reactivity of DBA decreases soon upon infection and maintains this trend during the disease progression period (r = 0.91) reaching more than 50% of reduction (P≤0.01) already at 18 days after infection and 78.8% of reduction (P<0.01) at the terminal stage (59 days). This consistent reduction of the glycans recognized by SBA, UEA-I, and DBA would probably disturb proteins stability for example by increasing the exposure of sites vulnerable to cleavage and denaturation.

These findings strongly suggest a potential role for mannose sugar recognized by ConA in the stabilization process, not only of the PrP but also of all the blood glycoproteins, occurring in hamster blood upon scrapie infection. This suggested role is also supported by the fact that a higher content of mannose sugar was previously detected in PrP^Sc^ from 263K-infected hamster brain [20] and in factor VI11 purified from human blood plasma and culture media of recombinant baby hamster kidney cells [21].

Consistent differences among the basal immunoreactivity (before infection) of the seven lectins toward plasma total proteins were observed (Figure 3(d)). These differences were maintained also after the scrapie infection. The basal immunoreactivity of ConA and RCA is 7.5- and 11.4-fold, respectively, higher than that of DBA, 10.2- and 15.6-fold, respectively, higher than that of SBA, 23.3- and 35.7-fold, respectively, higher than that of UEA-I. This may suggest that the type of sugars, their basal amount before infection, and the trend of alteration toward an increase or a decrease in their amount or exposure after infection could be considered as potential indicators/predictors of their inclination to be implicated in stabilizing, destabilizing or no action toward hamster blood glycoproteins including PrP,* in vivo*.

Calculation of the amount of the increase in the immunoreactivity of ConA and the decrease in the immunoreactivity of DBA, SBA, and UEA-I, after normalization against the basal immunoreactivity, revealed the existence of a correlation between the increase in ConA reactivity and the sum of the decrease in the reactivity of the three lectins (Figure 3(e)). This finding strongly suggests that the *α*–linked mannose recognized by ConA might be implicated in a stabilizing effect while the *α*-N-acetylgalactosamine, *α*-D-N-acetylgalactosamine, and (*α*-1, 2) linked fucose sugars recognized by DBA, SBA, and UEA-I, respectively, might be implicated in a destabilizing effect, toward blood glycoproteins after prion infection. Interestingly, the two opposite effects appear to be proportional (in quantitative terms) between each other (Figure 3(e)) suggesting that a compensatory mechanism might have been activated* in vivo*.

Based on these results, perhaps the glycoform of PrP^C^ that is greatly prone to convert into PrP^Sc^ is the glycoform highly disturbed its stability state by the prion infection. This conclusion is in accordance with an earlier study stating that a substantial portion of PrP^C^ is converted into PrP^Sc^ in cells in which the glycosylation machinery has been perturbed [11]. Such a glycoform of PrP^C^ would subsequently acquire major stability than a glycoform subjected to a minor destabilizing effect. Thus, PrP^C^ glycoform that is highly destabilized by prion infection and then stabilized is that glycoform that might efficiently convert into PrP^Sc^, at least for what concern the scrapie infection in hamsters. This is consistent with an earlier study in scrapie-infected neuroblastoma cells in which it has been demonstrated that the high mannose PrP^C^ glycoform is that preferentially converted into PrP^Sc^ [22].

Because of the minor destabilization occurred to ws-PrP^C^, which is for this reason less stable than classical PrP^C^, ws-PrP^C^ theoretically requires the involvement of few glycans to restore its stability state when is disturbed and therefore its propensity to convert into ws-PrP^Sc^ would be proportionally to this modest glycosylation alteration very low.

## 4. Conclusions

Little is known about the precise role of the N-linked glycosylation in the pathogenesis of prion diseases. This study provides the first characterization of the glycosylation profile of water soluble PrP^Sc^ using lectins and two different anti-PrP antibodies. Lectin-based assays are widely and reliably used for studying and characterizing glycosylation of proteins including PrP [15, 16, 23–25]. Lectins specifically bind to or cross-link carbohydrates on the surface of proteins [26, 27] enabling thus to profile cell-surface glycans and to correlate global changes in their expression with developmental stages and disease [28]. In addition, 3F4 and 6H4 are well characterized monoclonal antibodies that bind strongly and specifically to hamster PrP and are reactive to native and denatured forms of PrP [29–31].

In this study there have been differences between the wsPrP and classical PrP glycosylation profile, where glycans in ws-PrP were present in minor quantity and differently exposed. Analysis of the glycosylation profile of both ws-PrP and classical PrP revealed that these differences are responsible for the minor stability of wsPrP compared to classical PrP.

This ws-PrP instability caused by reduced glycosylation may have negatively influenced the propensity of ws-PrP^C^ to convert to ws-PrP^Sc^ both in blood and the brain.

There is a strong controversy regarding the glycosylation state of PrP^C^ and the efficiency of conversion to PrP^Sc^. Some studies have suggested that the glycosylation of PrP^C^ impedes the transmission between host species [32], whereas others have shown no such effect [33, 34].

Results obtained here are in accordance with the second opinion and suggest that the low glycosylation level of ws-PrP would refrain the conversion process* in vivo*. Accordingly, low amounts of ws-PrP^Sc^ were detected in hamster blood and brain [3] as to indicate the low efficiency of conversion of ws-PrP^C^ in the both compartments.

The efficiency of the conversion event in brain has recently been shown to be dependent on alteration in the PrP^C^ glycosylation status (glycoform type) [35]. Weisman et al. demonstrated that changing the glycosylation status of the host PrP^C^ can have profound effects on the magnitude of disease transmission. These findings, by Weisman group, suggest that conversion is dependent on the final glycoform produced by PrP^C^ glycosylation alteration and that not all glycoforms are able to convert into PrP^Sc^. Therefore, it is possible that the glycoform of hamster ws-PrP^C^, besides being unstable (susceptible to degradation), is also less prone to convert than the glycoform of classical PrP^C^. Besides these two limits, it might also be that there is a threshold limit under which the conversion process does not occur.

Thus, the PrP^C^/ws-PrP^C^ glycosylation magnitude and profile could be a key factor in determining risks of prion transmission from blood to brain (within different prion strains and species) and from one species to another. Furthermore, based on a recent assumption that factors intrinsic and extrinsic to blood may negatively affect the concentration and/or detection of PrP^Sc^/prions in blood in comparison to other tissues hampering thus the development of a routine blood test for prion diseases [19], ws-PrP^Sc^ glycosylation-related instability (not only* in vivo* but also* in vitro*) could be one of the main factors responsible for this delay. Therefore, an increase in the stability of ws-PrP^Sc^ by using GdnHCl may improve its quantitative and qualitative detection in blood at its actual concentration increasing consequently the possibility of accelerating the prion diseases diagnosis using blood.

In the current work, GdnHCl increased the stability of ws-PrP in hamster blood possibly by an interaction with its glycosylation profile. Preliminary analysis of the nature of this interaction by performing a global analysis of the lectin binding profile to ws-PrP^Sc^, classical PrP^Sc^ and plasma total proteins provided evidence on the potential involvement of some glycans in the stabilization process of glycoproteins that is supposed to occur in hamster blood following infection with scrapie and also allowed to reveal that glycans properties may be able to predict their potential role in this process.

This study may present some potential limitations that mainly concern the quality of the collected blood samples. In this respect, it has been observed that blood from terminally 263K-infected hamsters is more viscous and clots more quickly forming fibrin clots than blood from noninfected hamsters. If this would occur, the possible entrapment of ws-PrP and other proteins in the fibrin clots would interfere with their detection. Also, the quality of plasma samples represents a potential limitation since it can be affected by storage at room temperature and at 4°C over 24 hours and by repeated freeze/thaw cycles [36–38]. During this study it has been observed that protein degradation occurs and is evident after only 8 hours or less of storage at room temperature. Another important potential limitation is the formation of loosely pellets during the treatment with GdnHCl as stabilizing agent. If this pellet would not be separated slowly and accurately, then some high-abundance proteins would be released back into the water soluble fraction. Finally, treatment with proteinase K (PK) would impede the performance of a correct characterization of the ws-PrP using lectins, due to the release of new molecules of PrP from large aggregates present in hamster S^HS^ and plasma induced by PK, as previously demonstrated [3].

Although additional immunological analyses and* in vivo* studies are needed to better elucidate the role of PrP glycosylation-related instability in prion transmissibility, these results could be representative for other misfolded proteins that are characterized by having different glycoforms, including the soluble form, such as amyloid beta in Alzheimer's disease.

## Data Availability

No special data were used to support this study.

## Abbreviations

PrP: Prion protein
ws-PrP: Water-soluble PrP
PrPSc: Disease-associated prion protein
PrPc: Cellular prion protein
SHS: High-speed supernatant
PHS: High-speed pellet
lectin-ELISA: Lectin-enzyme-linked immunosorbent assay
GdnHCl: Guanidine hydrochloride
ConA: Concanavalin A
WGA: Wheat germ agglutinin
RCA: * Ricinus communis* agglutinin
DBA: * Dolichos biflorus* agglutinin
PNA: Peanut agglutinin
SBA: Soybean agglutinin
UEA-1: * Ulex europaeus* agglutinin I
IgGs: Immunoglobulins.
